# Identification of Nuclear Localization Signals in the ORF2 Protein of Porcine Circovirus Type 3

**DOI:** 10.3390/v11121086

**Published:** 2019-11-22

**Authors:** Chunxiao Mou, Minmin Wang, Shuonan Pan, Zhenhai Chen

**Affiliations:** 1 College of Veterinary Medicine, Yangzhou University, Yangzhou 225009, China; ytqxmcx@163.com (C.M.); psn_2017@163.com (S.P.); 2 Jiangsu Co-Innovation Center for Prevention and Control of Important Animal Infectious Diseases and Zoonoses, Yangzhou University, Yangzhou 225009, China; 3 Joint International Research Laboratory of Agriculture and Agri-Product Safety, the Ministry of Education of China, Yangzhou University, Yangzhou 225009, China

**Keywords:** porcine circovirus type 3, ORF2 protein, nuclear localization signal

## Abstract

Porcine circovirus type 3 (PCV3) contains two major open reading frames (ORFs) and the ORF2 gene encodes the major structural capsid protein. In this study, nuclear localization of ORF2 was demonstrated by fluorescence observation and subcellular fractionation assays in ORF2-transfected PK-15 cells. The subcellular localization of truncated ORF2 indicated that the 38 N-terminal amino acids were responsible for the nuclear localization of ORF2. The truncated and site-directed mutagenesis of this domain were constructed, and the results demonstrated that the basic amino acid residues at positions 8–32 were essential for the strict nuclear localization. The basic motifs _8_RRR-R-RRR_16_ and _16_RRRHRRR_22_ were further shown to be the key functional nucleolar localization signals that guide PCV3 ORF2 into nucleoli. Furthermore, sequence analysis showed that the amino acids of PCV3 nuclear localization signals were highly conserved. Overall, this study provides insight into the biological and functional characteristics of the PCV3 ORF2 protein.

## 1. Introduction

Porcine circoviruses (PCVs) are members of the family *Circoviridae* and the smallest DNA viruses [1]. Before 2015, porcine circovirus 1 (PCV1) and PCV2 were considered to be the only porcine circoviruses [2]. PCV1 is a cell-culture-derived virus and is nonpathogenic for swine. PCV2 is the primary etiological agent of porcine circovirus-associated diseases (PCVAD) that cause severe losses in the swine industry worldwide [3]. Recently, a novel circovirus was identified by next-generation sequence (NGS) analysis of aborted fetuses of sows and named PCV3. PCV3 was first reported in the USA in 2016 [4]. Since then, PCV3 has been detected in many countries, and it is associated with porcine dermatitis and nephropathy syndrome, congenital tremors, reproductive failure, and multi-systemic inflammation [5,6,7,8].

Circoviruses are the smallest autonomously replicating DNA viruses, and they show a typical ambisense genomic structure [9,10]. Circoviruses have a non-enveloped, circular, single-stranded DNA genome consisting of two major open reading frames, ORF1 and ORF2, which code the replicase (Rep) and the capsid protein (Cap), respectively [11,12]. As in PCV1 and PCV2, PCV3 *ORF2* encodes the only structural capsid protein, which contains the dominant immunological regions [11,13]. Circoviruses lack an autonomous DNA polymerase and are dependent on the replication machinery of the host cell for de novo DNA synthesis. As DNA synthesis occurs exclusively in the nucleus, the active nuclear import of DNA molecules might require the involvement of karyophilic proteins [14]. In the case of PCVs, the N-terminus of ORF2 is rich in basic amino acids and displays nuclear localization signals (NLSs) [14,15,16]. The NLS is a short stretch of amino acids that mediates the transport of nuclear proteins into the nucleus. NLS motifs play a key role in this mechanism. NLS sequences are often composed of basic amino acids and can be classified as either monopartite or bipartite motifs [17,18]. The nuclear targeting of PCV2 ORF2 is directed by the bipartite motifs situated at the N-terminus of the proteins [14,15]. NLSs of PCV1 ORF2 show high homologies to classical monopartite or bipartite NLS, which is essential for the complete nuclear import of PCV1 ORF2. The N-terminus of PCV1 and PCV2 ORF2 contain several conserved basic amino acid stretches [15,19], and shares 70.7% in nucleotide identity and 82.9% in amino acid similarity.

Verification of NLS and demonstration of intracellular distribution facilitate understanding of viral protein function. The amino acids of PCV3 ORF2 are markedly different from those of the other PCVs. This led us to study the functional motifs in nuclear targeting of PCV3 ORF2. In this study, a series of recombinant plasmids expressing PCV3 ORF2 fused to EGFP were constructed, and we identified the main motifs of the NLSs mediating nuclear localization of PCV3 ORF2.

## 2. Materials and Methods

### 2.1. Plasmids

The PCV3 GD-HZ (GenBank accession number: MK454953.1) ORF2 gene and its truncated fragments were amplified using the High-Fidelity PCR System (TaKaRa). Primer annealing was used to obtain ORF2-1-1–16 fragments, and ORF2-∆NLS 2,3, ORF2-∆NLS 1,2, ORF2-∆NLS 1,3, and ORF2-∆NLS 1,2,3 fragments were amplified using PCR. Then, all fragments were inserted into pEGFP-C3 (Clontech, Takara Bio, Kusatsu, Japan) to get recombinant plasmids. All primers and oligonucleotides used are shown in Table 1.

### 2.2. Cells and Antibodies

PK-15 cells free of PCV (ATCC CCL33; American Type Culture Collection, Rockville, MD, USA) were cultured, at 37 °C and 5% CO_2_, in DMEM (Gibco, Carlsbad, CA, USA) supplemented with 10% fetal bovine serum. Anti-GFP antibody and anti-GAPDH antibody were obtained from Bioss (Beijing, China). Horseradish peroxidase (HRP)-conjugated goat anti-mouse IgG antibody was purchased from Santa Cruz Biotechnology (Santa Cruz, CA, USA).

### 2.3. Transfection and Fluorescence Analysis

PK-15 cells were grown on tissue culture plates for 24 h, and 70–80% confluent cells were transfected with the recombinant plasmids using the Lipofectamine 3000 transfection reagent (Invitrogen, Carlsbad, CA, USA) according to the manufacturer’s instructions. To analyze the localization of the expressed proteins, PK-15 cells were fixed with 4% paraformaldehyde for 30 min at 4 °C, then washed with PBS, and the nucleus was stained with 4′,6′-diamidino-2-phenylindole (DAPI). Fluorescence was examined under a laser confocal scanning microscope.

### 2.4. Nuclear and Cytoplasmic Protein Extraction

PK-15 cells grown into six well plates, were transfected with the recombinant plasmids and incubated at 37 °C for 24 h. The nuclear and cytoplasmic protein were extracted using the Nuclear and Cytoplasmic Protein Extraction Kit (Sangon Biotech, Shanghai, China) following the manufacturer’s protocol. The cells were then washed gently with PBS and collected. The cell pellets were resuspended in 100 μL cytoplasmic protein extraction buffer A, incubated on ice for 3 min, and then the preparation was spun at 1500× *g* for 4 min. The supernatants were transferred as cytoplasmic protein, and the nuclear pellets were resuspended using 50 μL nuclear protein extraction buffer B. We then incubated the mixture on ice for 10 min, and it was swirled to resuspend the pellets. The nuclear protein extraction was centrifuged at 12,000× *g* for 10 min, and the supernatants were collected as the nuclear protein.

### 2.5. Western Blot

After transfection, cells were lysed with 5× SDS sample buffer and boiled for 10 min before loading onto a 12% SDS-PAGE gel. Proteins were transferred to membranes, followed by incubation with mouse anti-GFP antibody (1:2000) and anti-GAPDH antibody (1:5000). Membranes were then incubated with HRP-conjugated goat anti-mouse IgG antibody at 1:5000 dilution. Blots were visualized using Image-Pro software.

### 2.6. Statistical Analysis

GraphPad Prism software (GraphPad Software, San Diego, CA, USA) was used for data analysis. Data from three independent experiments were shown as the mean ± standard error (SE). The differences between groups were determined by one-way ANOVA.

## 3. Results

### 3.1. Localization of NLSs in ORF2

**Figure 1:**
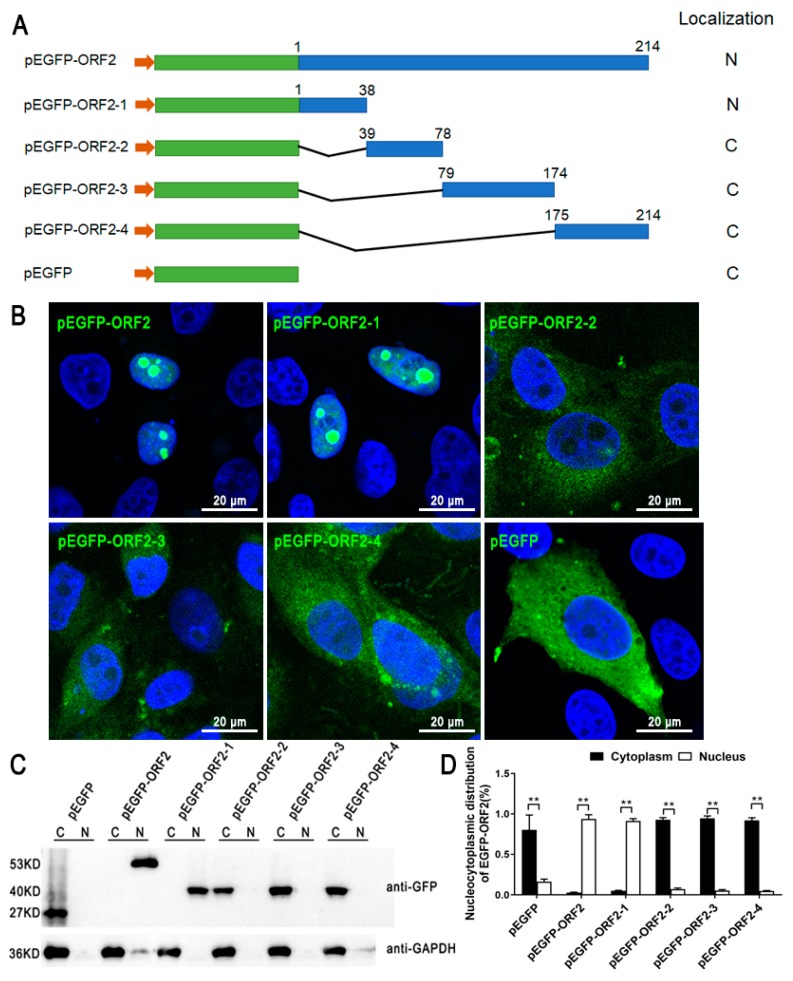
The localization of nuclear localization signals (NLSs) in ORF2. (**A**) Recombinant plasmids containing ORF2 or truncated ORF2 fragments with EGFP in the N-terminus were constructed. The subcellular localization of fusion proteins is indicated by N (nuclear) or C (cytoplasmic). (**B**) The localization of fusion proteins in transfected cells was observed by confocal microscopy. Scale bars = 20 μm. (**C**) and (**D**) Transfected cells were subjected to nuclear and cytoplasmic extraction. The abundance of expressed proteins in the nuclear and cytoplasmic extracts were detected by western blot. Nuclear/cytoplasmic distribution of the expressed proteins was further analyzed through densitometric quantification using Image-Pro software, data from three independent experiments are shown on the graph as the average ± standard error. One-way ANOVA; ** *p* < 0.01.

**Figure 2:**
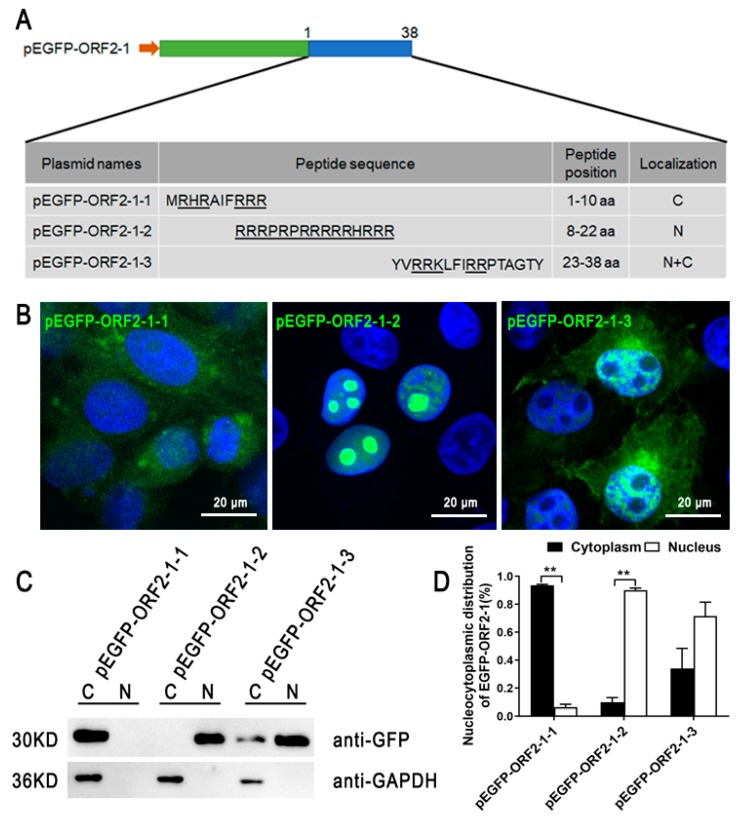
The NLS motifs in ORF2-1. (**A**) pEGFP-ORF2-1-1 (1–10 aa), pEGFP-ORF2-1-2 (8–22 aa), pEGFP-ORF2-1-3 (23–38 aa) were constructed and predicted NLSs were listed. (**B**) PK-15 cells were transfected with recombinant plasmids and observed by confocal microscopy after 24 h. Scale bars = 20 μm. (**C**) and (**D**) The nucleus and cytoplasm were extracted from the transfected PK-15 cells, and the abundance of expressed proteins in the extracts were detected by western blot after nuclear and cytoplasmic extraction, nuclear/cytoplasmic distribution of the expressed proteins was analyzed through densitometric quantification using Image-Pro software, data from three independent experiments are shown on the graph as the average ± standard error. One-way ANOVA; ** *p* < 0.01.

To confirm the NLS activity of PCV3 ORF2, the ORF2 gene was inserted into pEGFP-C3. EGFP-ORF2 was completely located in the nucleus of PK-15 cells, and EGFP alone displayed a diffuse cytoplasmic distribution (Figure 1B). To determine the important motifs of the ORF2 in its nuclear localization function, recombinant plasmids (pEGFP-ORF2-1, pEGFP-ORF2-2, pEGFP-ORF2-3, and pEGFP-ORF2-4) were generated and transferred into PK-15 cells. The mutants are shown in Figure 1A. EGFP-ORF2-1 could accumulate in the nucleus and EGFP-ORF2-2, EGFP-ORF2-3, and EGFP-ORF2-4 were located in cytoplasm (Figure 1B–D). This implied that the N-terminal residues (1–38 aa) of ORF2 play a role in nuclear localization. Three truncation mutants were constructed from ORF2-1 (Figure 2A). Figure 2B–D shows that EGFP-ORF2-1-2 is located in the nucleus and mostly in the nucleoli. EGFP-ORF2-1-3 was present in both the nucleus and the cytoplasm.

### 3.2. Key Residues of NLSs in ORF2

**Figure 3:**
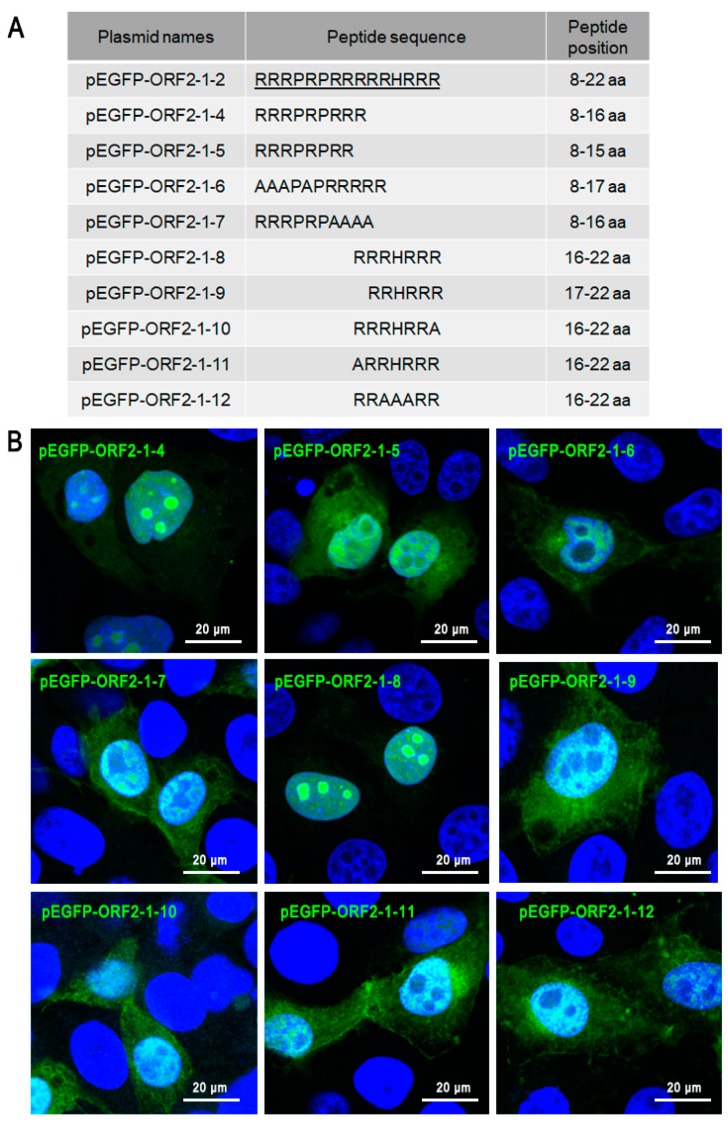
The main nucleolar localization signal motifs in ORF2-1-2. (**A**) Mutants of ORF2-1-2 gene were inserted into pEGFP-C3 and predicted nucleolar localization signal motifs are underlined. (**B**) PK-15 cells were transfected with plasmids, after 24 h, the cells were fixed and the localization of fusion proteins was observed by confocal microscopy. Scale bars = 20 μm.

**Figure 4:**
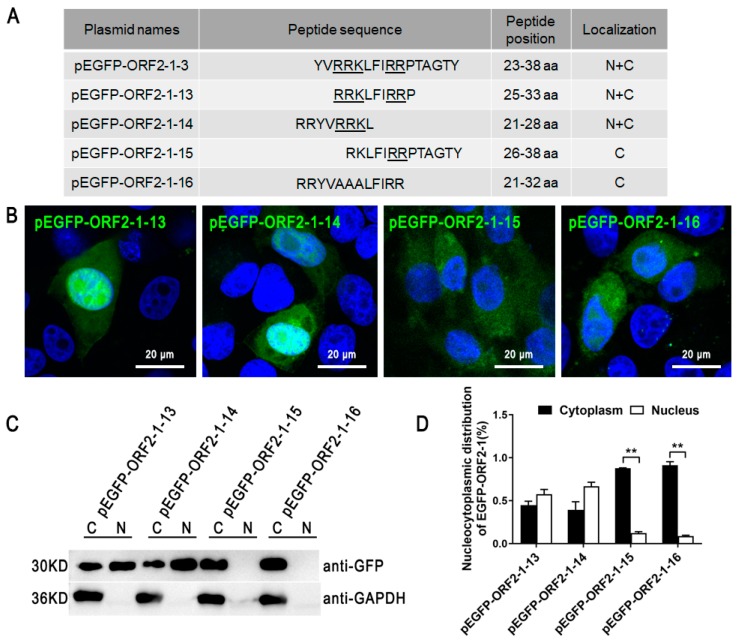
The key amino acids of NLS in ORF2-1-3. (**A**) Mutants of ORF2-1-3 gene were inserted into pEGFP-C3, and the key amino acids predicted in ORF2-1-3 are listed by underline. (**B**) PK-15 cells were transfected with mutant plasmids, and the localization of fusion proteins was observed by confocal microscopy. Scale bars = 20 μm. (**C**) and (**D**) After 24 h, the nucleus and cytoplasm in transfected cells were extracted and the expressed proteins were determined by western blot. Nuclear/cytoplasmic distribution of the expressed proteins was further analyzed through densitometric quantification using Image-Pro software, data from three independent experiments are shown on the graph as the average ± standard error. One-way ANOVA; ** *p* < 0.01.

To study the vital basic amino acids of the ORF2-1-2 (8–22 aa) motif in its nucleolar localization function, truncated versions of the ORF2-1-2 fragment were cloned using pEGFP-C3 (Figure 3A). EGFP-ORF2-1-4 (8–16 aa) and EGFP-ORF2-1-8 (16–22 aa) were mostly localized in the nucleoli, while EGFP-ORF2-1-5 (8–15 aa) and EGFP-ORF2-1-9 (17–22 aa) were located in the nucleus (Figure 3B). Single and multi-nucleotide alanine (A) substitution mutants of the ORF2-1-2 fragment were constructed using pEGFP-C3 (Figure 3A). The results showed ORF2-1-6, ORF2-1-7, ORF2-1-10, ORF2-1-11, and ORF2-1-12 mutants completely limited the function of nucleoli localization, suggesting that _8_RRR-R-RRR_16_ and _16_RRRHRRR_22_ were the two main nucleolar localization motifs in ORF2-1-2. We studied the vital basic amino acids of ORF2-1-3 (23–38 aa) by constructing variants using truncation and site-directed mutagenesis (Figure 4A). EGFP-ORF2-1-13, and EGFP-ORF2-1-14 displayed similar nucleo-cytoplasmic distribution (Figure 4B–D). However, EGFP-ORF2-1-15 and EGFP-ORF2-1-16 were exclusively localized in the cytoplasm. These results demonstrated that _21_RR_22_, _25_RRK_27_, and _31_RR_32_ in ORF2 (23–38 aa) were the key residues of nuclear localization in ORF2-1-3.

### 3.3. Map of NLSs in ORF2

**Figure 5:**
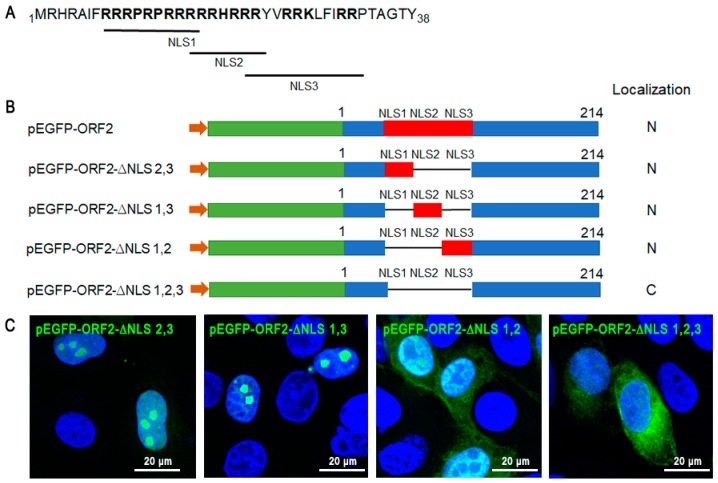
The map of NLSs in ORF2. (**A**) The main motifs of the NLSs in the N-terminal ORF2 (1–38 aa) are bold and the three NLSs are listed. (**B**) A sketch of the ORF2 indicating putative NLSs motifs by red boxes is shown, and the mutated fragments of the ORF2 are shown. (**C**) PK-15 cells were transfected with plasmids carrying mutated ORF2 gene (pEGFP-ORF2-∆NLS 2,3, pEGFP-ORF2-∆NLS 1,2, pEGFP-ORF2-∆NLS 1,3, and pEGFP-ORF2-∆NLS 1,2,3) to analyze their subcellular location. Subcellular localization of green fusion proteins was analyzed by confocal microscopy. Scale bars = 20 μm.

There were three NLS motifs in PCV3-ORF2: NLS1 (8–16 aa), NLS2 (16–22 aa), and NLS3 (21–32 aa), and the key residues are shown in bold in Figure 5A. To define the contribution of each NLS motif in its nuclear localization function, individual NLS motifs or all of them were deleted (Figure 5B). EGFP-ORF2-∆NLS2,3 and EGFP-ORF2-∆NLS1,3 could be localized in the nucleoli, EGFP-ORF2-∆NLS1,2 displayed almost equal nucleo-cytoplasmic distribution, and EGFP-ORF2-∆NLS1,2,3 was localized in the cytoplasm (Figure 5C). These data indicated that NLS1 (8–16 aa) and NLS2 (16–22 aa) were critical for the nuclear localization of ORF2, and NLS3 (21–32 aa) could not lead strict nucleus localization.

### 3.4. Variability of PCV3 ORF2 NLSs

**Figure 6:**
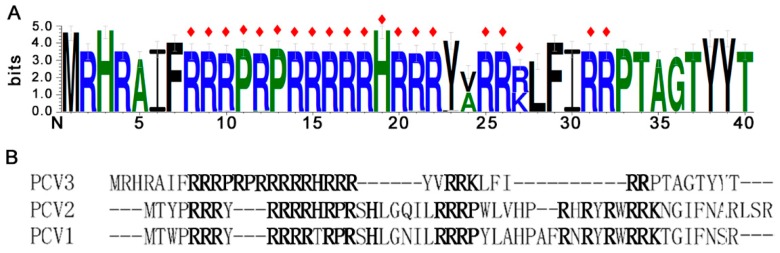
The variability of PCV3-ORF2 NLSs. (**A**) Logo of the N-terminal of all PCV3 ORF2 proteins (1–40 aa) reported and their complementary sequences, the nuclear localization signal peptide is showed by red ◊. (**B**) The NLSs sequence alignment between PCV3 ORF2 and other PCVs is showed and the key amino acids are bold.

To study the variability of NLSs, the logo of the N-terminal region in all PCV3 ORF2 sequences was created through http://weblogo.threeplusone.com/create.cgi. The NLS region sequence alignment logo showed that only lysine 27 was substituted by the basic amino acid arginine in some strains (Figure 6A). The NLSs sequence alignment between PCV3 ORF2 and other PCVs was created through https://www.ebi.ac.uk/Tools/msa/clustalo/. The result showed that the NLSs motifs identified in PCVs were similar, especially, the amino acids of nucleolar localization signals (Figure 6B).

## 4. Discussion

Since 2016, PCV3 has been reported in at least seven countries [4]. PCV3 is associated with porcine dermatitis and nephropathy syndrome, congenital tremors, reproductive failure, and multi-systemic inflammation. PCV3 infection in piglets triggers inflammatory lesions in various tissues and organs followed by lymphocytic dysplasia and necrosis, and disruption of the immune system [20]. However, the exact pathogenesis of PCV3 remains unclear [21,22,23]. The genome of PCV3 contains two major open reading frames (ORFs): ORF1 encoding replicase and ORF2 encoding capsid [24]. The capsid protein is a karyophilic protein located in the nucleus [14,16]. We found that PCV3 ORF2 could also target the nucleus. Sequence analysis indicated that the N-terminus of PCV3 ORF2 contained many conserved basic amino acids. Investigation of subcellular localization of truncated PCV3 ORF2 fused with EGFP showed that the 38 amino acids at the N-terminus were necessary and sufficient to direct the accumulation of protein in nucleus. This is similar to PCV1 and PCV2 ORF2 [15,19].

Even though there is no strict consensus on NLS, other NLS sequences are generally divided into classical monopartite NLS and classical bipartite NLS [25]. To study the essential NLS motifs at the N-terminal of PCV3 ORF2, truncations or substitutions of basic amino acids were introduced into these stretches. Three NLS motifs in PCV3-ORF2 (NLS1, NLS2, and NLS3) were identified. NLS1 (_8_RRR-R-RRR_16_) and NLS2 (_16_RRRHRRR_22_) were two contiguous motifs. These stretches showed homology to the “pat4” motif consisting of four continuous basic residues, or the “bipartite” motif that contains two stretches of basic amino acids segregated by non-conserved residues [26]. The similar motifs were also identified in PCV1 ORF2 (_4_PRRR-RRRR-RPR-H_18_) and PCV2 ORF2 (_4_PRRR-RRRRHRPR_18_) [15,19]. The subcellular fractionation results showed that NLS1 and NLS2 could direct strict nucleus localization, which was similar to the full-length PCV3 ORF2. The other “pat4” motif NLS3 fused with EGFP displayed almost equal nucleo-cytoplasmic distribution. These data suggest that NLS1 and NLS2 are the key NLSs in the nuclear localization of PCV3 ORF2 and that NLS3 plays an auxiliary role.

DNA synthesis of circoviruses occurs exclusively in the nucleus of host cells, but the active nuclear import of DNA molecules might require karyophilic proteins [14]. In the case of circoviruses, such as PCVs and beak and feather disease virus (BFDV), the NLS region of is important for ssDNA accumulation [14,16]. The N-terminal of PCV2 ORF2 can interact with the nuclear membrane receptor (gC1qR) to regulate DNA [27]. This suggests that the NLS region of PCV3 ORF2 may be involved in DNA binding. In addition, PONDR analysis predicted that the N-terminal of PCV2 ORF2 was a disordered peptidic region lacking a well-defined 3D structure under physiological conditions [27]. This arginine-rich region has a high probability of being exposed to solvent and then interacting with surrounding proteins [28]. The NLS region may contain some dominant epitopes, and these epitopes may influence the antigenicity of Cap. Further study is needed to verify whether there are dominant epitopes in the NLS region of PCV3 ORF2.

Nucleolar localization has been described for the proteins of many DNA and RNA viruses [29]. These viral proteins play multifunctional roles in regulating cellular transcription [30,31], virus transcription [32], virus translation [33], and cell division [34]. For example, hepatitis delta virus (HDV) was studied for nucleolar localization and it was found that interacting with nucleolin promoted viral replication [35]. PCV1 ORF2 was localized in the nucleoli during PCV1 early infection, followed by co-localization with Rep in the nucleoplasm [36]. In addition, the PCV1 and PCV2 ORF2 might help regulate viral replication by interacting with Rep [37]. These suggest that the nucleolar localization signals in PCV3 ORF2 might be involved in the regulation of viral replication. Further studies will be needed to detail the function of nucleolar localization signals. The findings will clarify the function of PCV3 ORF2 in the viral replication and pathogenicity.

## Figures and Tables

**Table 1:** Oligonucleotides.

Name	Oligonucleotide Sequence in 5′–3′ Direction	Restriction Site
pEGFP-ORF2 F	CTCAAGCTTATGAGACACAGAGCTATATT	*Hind*III
pEGFP-ORF2 R	GGTGGATCCTTAGAGAACGGACTTGTAAC	*Bam*HI
pEGFP-ORF2-1 R	GGTGGATCCGTATGTGCCAGCTGTGGGCC	*Bam*HI
pEGFP-ORF2-2 F	CTCAAGCTTTACACAAAGAAATACTCCAC	*Hind*III
pEGFP-ORF2-2 R	GGTGGATCCAAAGCTAATGGCAGTTTCCC	*Bam*HI
pEGFP-ORF2-3 F	CTCAAGCTTGAATATTATAAGATACTAAA	*Hind*III
pEGFP-ORF2-3 R	GGTGGATCCGTTGAGCCATGGGGTGGGTC	*Bam*HI
pEGFP-ORF2-4 F	CTCAAGCTTACATATGACCCCACCGTTCA	*Hind*III
pEGFP-ORF2-1-1 F	AGCTTATGAGACACAGAGCTATATTCAGAAGAAGAG	*Hind*III
pEGFP-ORF2-1-1 R	GATCCTCTTCTTCTGAATATAGCTCTGTGTCTCATA	*Bam*HI
pEGFP-ORF2-1-2 F	AGCTTAGAAGAAGACCCCGCCCAAGGAGGCGACGACGCCACAGAAGGCGCG	*Hind*III
pEGFP-ORF2-1-2 R	GATCCGCGCCTTCTGTGGCGTCGTCGCCTCCTTGGGCGGGGTCTTCTTCTA	*Bam*HI
pEGFP-ORF2-1-3 F	AGCTTTATGTCAGAAGAAAACTATTCATTAGGAGGCCCACAGCTGGCACATACG	*Hind*III
pEGFP-ORF2-1-3 R	GATCCGTATGTGCCAGCTGTGGGCCTCCTAATGAATAGTTTTCTTCTGACATAA	*Bam*HI
pEGFP-ORF2-1-4 F	AGCTTAGAAGAAGACCCCGCCCAAGGAGGCGAG	*Hind*III
pEGFP-ORF2-1-4 R	GATCCTCGCCTCCTTGGGCGGGGTCTTCTTCTA	*Bam*HI
pEGFP-ORF2-1-5 F	AGCTTAGAAGAAGACCCCGCCCAAGGAGGG	*Hind*III
pEGFP-ORF2-1-5 R	GATCCCCTCCTTGGGCGGGGTCTTCTTCTA	*Bam*HI
pEGFP-ORF2-1-6 F	AGCTT**GCAGCAGCA**CCC**GCA**CCAAGGAGGCGACGAG	*Hind*III
pEGFP-ORF2-1-6 R	GATCCTCGTCGCCTCCTTGG**TGC**GGG**TGCTGCTGC**A	*Bam*HI
pEGFP-ORF2-1-7 F	AGCTTAGAAGAAGACCCCGC**GCAGCAGCAGCAGCA**G	*Hind*III
pEGFP-ORF2-1-7 R	GATCC**TGCTGCTGCTGCTGC**GCGGGGTCTTCTTCTA	*Bam*HI
pEGFP-ORF2-1-8 F	AGCTTCGACGACGCCACAGAAGGCGCG	*Hind*III
pEGFP-ORF2-1-8 R	GATCCGCGCCTTCTGTGGCGTCGTCGA	*Bam*HI
pEGFP-ORF2-1-9 F	AGCTTCGACGCCACAGAAGGCGCG	*Hind*III
pEGFP-ORF2-1-9 R	GATCCGCGCCTTCTGTGGCGTCGA	*Bam*HI
pEGFP-ORF2-1-10 F	AGCTTCGACGACGCCACAGAAGG**GCA**G	*Hind*III
pEGFP-ORF2-1-10 R	GATCC**TGC**CCTTCTGTGGCGTCGTCGA	*Bam*HI
pEGFP-ORF2-1-11 F	AGCTT**GCA**CGACGCCACAGAAGGCGCG	*Hind*III
pEGFP-ORF2-1-11 R	GATCCGCGCCTTCTGTGGCGTCG**TGC**A	*Bam*HI
pEGFP-ORF2-1-12 F	AGCTTCGACGA**GCAGCAGCA**AGGCGCG	*Hind*III
pEGFP-ORF2-1-12 R	GATCCGCGCCT**TGCTGCTGC**TCGTCGA	*Bam*HI
pEGFP-ORF2-1-13 F	AGCTTAGAAGAAAACTATTCATTAGGAGGCCCG	*Hind*III
pEGFP-ORF2-1-13 R	GATCCGGGCCTCCTAATGAATAGTTTTCTTCTA	*Bam*HI
pEGFP-ORF2-1-14 F	AGCTTAGGCGCTATGTCAGAAGAAAACTAG	*Hind*III
pEGFP-ORF2-1-14 R	GATCCTAGTTTTCTTCTGACATAGCGCCTA	*Bam*HI
pEGFP-ORF2-1-15 F	AGCTTAGAAAACTATTCATTAGGAGGCCCACAGCTGGCACATACG	*Hind*III
pEGFP-ORF2-1-15 R	GATCCGTATGTGCCAGCTGTGGGCCTCCTAATGAATAGTTTTCTA	*Bam*HI
pEGFP-ORF2-1-16 F	AGCTTAGGCGCTATGTC**GCAGCAGCA**CTATTCATTAGGAGGG	*Hind*III
pEGFP-ORF2-1-16 R	GATCCCCTCCTAATGAATAG**TGCTGCTGC**GACATAGCGCCTA	*Bam*HI
pEGFP-ORF2-∆NLS 2,3 F1	AAGACCCCGCCCAAGCCCACAGCTGGCACATAC	
pEGFP-ORF2-∆NLS 2,3 F2	GAGACACAGAGCTATATTCAGAAGAAGACCCCGCCCAAG	
pEGFP-ORF2-∆NLS 1,3 F1	CGACGACGCCACAGAAGGCGCCCCACAGCTGGCACATAC	
pEGFP-ORF2-∆NLS 1,3 F2	CACAGAGCTATATTCCGACGACGCCACAGA	
pEGFP-ORF2-∆NLS 1,2 F	CACAGAGCTATATTCAGGCGCTATGTCAGA	
pEGFP-ORF2-∆NLS 1,2,3 F	CACAGAGCTATATTCCCCACAGCTGGCACATAC	

The nucleotides of restriction sites are underlined and mutants are bolded.
